# A tree-based corpus annotated with Cyber-Syndrome, symptoms, and acupoints

**DOI:** 10.1038/s41597-024-03321-0

**Published:** 2024-05-10

**Authors:** Wenxi Wang, Zhan Zhao, Huansheng Ning

**Affiliations:** School of Computer & Communication Engineering, University of Science and Technology Beijing, Beijing, 100083 China

**Keywords:** Diseases, Therapeutics

## Abstract

Prolonged and over-excessive interaction with cyberspace poses a threat to people’s health and leads to the occurrence of Cyber-Syndrome, which covers not only physiological but also psychological disorders. This paper aims to create a tree-shaped gold-standard corpus that annotates the Cyber-Syndrome, clinical manifestations, and acupoints that can alleviate their symptoms or signs, designating this corpus as CS-A. In the CS-A corpus, this paper defines six entities and relations subject to annotation. There are 448 texts to annotate in total manually. After three rounds of updating the annotation guidelines, the inter-annotator agreement (IAA) improved significantly, resulting in a higher IAA score of 86.05%. The purpose of constructing CS-A corpus is to increase the popularity of Cyber-Syndrome and draw attention to its subtle impact on people’s health. Meanwhile, annotated corpus promotes the development of natural language processing technology. Some model experiments can be implemented based on this corpus, such as optimizing and improving models for discontinuous entity recognition, nested entity recognition, etc. The CS-A corpus has been uploaded to *figshare*.

## Background & Summary

Nowadays, with the rapid development of Information and Communication Technology (ICT), the term “cyberspace” has undoubtedly become an increasingly attractive space in our daily lives, work, and study^1^ Cyberspace is an abstract, virtual, digital space based on various infrastructures, equipment, software, and hardware. The emergency of cyberspace has modified how humans interact, reshaping traditional communication media such as cinema, television, music, and the telephone. In cyberspace, information can be exchanged regardless of time and space, and the information exchanged has the advantages of time-domain (fast update), interactivity (human-to-human and human-to-information interaction), diversity of forms (e.g. pictures, videos, voice, etc.), and outstanding personalization. As a result, it attracts many users to participate in it. People also spend more and more time in cyberspace. As the size of cyberspace users continues to expand and the time spent in cyberspace gradually increases, Cyber-Syndrome deserves the growing attention of more and more people.

In 2018, our group defined the concept and connotation of Cyber-Syndrome. Namely, Cyber-Syndrome is a physical, social, and mental disorder that affects humans due to misuse of technology or excessive interaction with cyberspace^2^ For example, the occurrence of *computer neck* due to prolonged low-headed use of electronic devices; *Computer spondylosis* is caused by continuous Internet access and improper posture for a significant period; Prolonged intensive, repetitive, and excessive finger movements can cause wrist joint paralysis and pain, increase the incidence of *Carpal Tunnel Syndrome*, etc. There are many types of Cyber-Syndrome and a variety of pathogenic factors, such as occupation, family environment, and personal habits that can catalyze the onset of Cyber-Syndrome. Cyber-Syndrome threatens people’s physical and mental health and can be life-threatening in severe cases. However, Cyber-Syndrome has not been systematically outlined, numerous scientific papers have experimentally demonstrated that prolonged or excessive Internet use can lead to Cyber-Syndrome, and electronic clinical records have shown that some Cybe-Syndrome causation is related to inappropriate interactions in cyberspace, and different Cyber-Syndrome have different clinical manifestations. Currently, These data and information exist in an unstructured form and a fragmented state.

Natural Language Processing (NLP) is a computer-aided analysis technique designed to automatically analyze and understand human language, enabling scholars to effortlessly extract valuable insights contained in textual data sets while avoiding heavy computational work^3^ However, Most NLP techniques (especially those based on machine learning algorithms) rely on annotated data sets, which can be used to train and test the proposed NLP models^4^ Therefore, we aim to create a golden corpus annotated with Cyber-Syndrome, signs, and symptoms. Scholars can use NLP techniques to extract relevant information, enrich the knowledge system of Cyber-Syndrome, and raise awareness of Cyber-Syndrome for better adaptation to cyberspace.

Besides, how to effectively treat Cyber-Syndrome is becoming a common problem for global cyberspace users. Disease treatment is divided into two perspectives: Traditional Chinese medicine (TCM) and Western medicine, which are two completely different systems, from the way of thinking to the treatment methods are not the same. Since Cyber-Syndrome is formed by long-term chronic accumulation, treatment with TCM is more suitable for the prevention and treatment of Cyber-Syndrome. The theory of meridians and acupoints is one of the fundamental theories of TCM. There are unique spots on the meridians that are neither isolated from the body’s surface but are closely connected and interconnected with deep tissues and organs, i.e. acupoints. On the one hand, it reflects disease and pain; on the other hand, it can undergo stimulation to prevent and treat disease. Not all diseases can be cured by acupoint therapy, but acupoints have a remarkable effect in alleviating the symptoms of diseases. Therefore, we established a tree-based corpus by acquiring a corpus of acupoints and automating the mapping of acupoints that can alleviate the symptoms of Cyber-Syndrome.

This paper integrates the disease corpus included in Cyber-Syndrome to construct a Cyber-Syndrome corpus and constructs an acupoint corpus to alleviate the Cyber-Syndrome symptoms from the perspective of TCM. The CS-A corpus connects the Cyber-Syndrome corpus and the acupoint corpus using the hierarchical structure of a tree, and manually annotates key entities such as Cyber-Syndrome, symptoms, and acupoints, as well as the relationships that exist between the entities. This paper presents the annotation process and guidelines in detail and consistently analyzes the annotation results with 86.05% agreement rate.

## Methods

### Data sources and preparation

#### Cyber-Syndrome

Prolonged or excessive interaction with cyberspace can easily result in physiological and psychological disorders of users, which we call Cyber-Syndrome. To facilitate the study of Cyber-Syndrome, we generalized the diseases belonging to Cyber-Syndrome from the published studies. We constructed a Cyber-Syndrome lexicon to prepare data for the pre-annotation of the CS-A corpus. We systematically searched the Science Citation Index Embase, PubMed, Web of Science, and other databases. By conducting the meta-analysis of clinical trial results, scholars are able to collect statistics and analyze a large number of individual studies to get more objective findings, which is the highest level of the evidence-based medicine hierarchy. Based on the meta-analysis results, we summarized the various diseases encompassed by Cyber-Syndrome, mainly divided into physiological and psychological disorders.

Physiological disorders are usually the result of a malfunction of a body organ, non-functioning or changes in the actual cellular structure over a while that cause illness. Psychological disorders are characterized by a clinically significant impairment in an individual’s cognition, emotional regulation, or behavior. It is usually associated with distress or impairment in critical areas of functioning. We compiled a list of physiological disorders (see Table 1) and a list of psychological disorders (see Table 2) encompassed by the Cyber-Syndrome through the results of a meta-analysis of the literature. To improve the consensus of Cyber-Syndrome internationally, a coarse-grained classification of physiological and psychological disorders of Cyber-Syndrome is made by utilizing the ICD-11 issued by the World Health Organization (i.e., the root nodes of various disorders in the ICD-11). Meanwhile, each file of the Cyber-Syndrome corpus was named using the unique code of MeSH (Medical Subject Headings), published by the National Library of Medicine (NLM), and is currently the most authoritative and commonly used standard medical subject headings.

**Table 1:** Physiological disorders caused by prolonged or excessive interaction with cyberspace.

ICD-11	Cyber-Syndrome	MeSH	Indicator		Ref
			OR/RR	95%CI	
Histopathology	Glioma	D005910	1.44	[1.08, 1.91]	^42^
			1.46	[1.12, 1.92]	
	Acoustic Neuroma	D009464	1.9	[0.90, 4.10]	^43^
			3.9	[1.60, 9.50]	
Diseases of the visual system	Myopia	D009216	1.77	[1.28, 2.45]	^44^
	Diabetic Retinopathy	D003930	1.18^*^	[1.01, 1.37]	^45^
Disease of the nervous system	Headache	D006261	1.38	[1.18, 1.61]	^46^
	Carpal Tunnel Syndrome	D002349	1.93	[1.43,2.61]	^47^
	Stroke	D020521	1.7	[1.23, 2.35]	^48^
Neoplasms	Uveal Melanoma	D014604	4.2	[1.20, 14.5]	^49^
	Brain Neoplasms	D001932	1.32	[1.03, 1.70]	^50^
			1.25	[1.02, 1.53]	
	Breast Neoplasms	D001943	1.77	[1.07, 2.92]	^51^
	Colorectal Neoplasms	D015179	1.25	[1.05, 1.49]	^52^
	Ovarian Neoplasms	D010051	1.29^*^	[1.07, 1.57]	^53^
	Thyroid Neoplasms	D065646	1.58^*^	[0.98, 2.54]	^54^
Eye, ear, nose and throat system disorders	Tinnitus	D014012	1.95	[1.00, 3.80]	^55^
Mental, behavioural or neurodevelopmental disorders	Dementia	D003704	1.3	[1.12,1.51]	^56^
Disease of the digestive system	Gallstones	D042882	1.15	[1.02, 1.29]	^57^
	Temporomandibular Joint Disorders	D013705	1.81	[1.08, 3.04]	^58^
Endocrine, nutritional or metabolic diseases	Diabetes Mellitus	D003920	1.47	[1.17, 1.84]	^48^
	Diabetes Mellitus, Type 2	D003924	1.20	[1.14, 1.27]	^59^
	Metabolic Syndrome	D024821	1.19	[1.10, 1.30]	^28^
	Xerophthalmia	D014985	1.55	[1.25, 1.91]	^60^
Diseases of the circulatory system	Deep vein thrombosis	D020246	1.35^*^	[1.07, 1.70]	^61^
	Pulmonary Embolism	D011655			
	Hypertension	D006973	1.27	[1.13, 1.42]	^48^
	Angina Pectoris	D000787	1.35	[1.23, 2.35]	
	Myocardial Infarction	D009203	1.47	[1.13, 1.90]	
	Cardiac Arrhythmia	D001145	1.34	[1.01, 1.78]	
Diseases of the respiratory system	Chronic Obstructive				
	Pulmonary Disease	D029424	1.40	[1.07, 1.85]	^48^
	Allergic Rhinitis	D065631	1.17	[1.11, 1.22]	^62^
	Asthma	D001249	1.23	[1.15, 1.32]	
No classification elsewhere	Heart Murmurs	D006337	1.39	[1.08, 1.79]	^48^

The Odds ratio (OR), in case-control studies, is the ratio of the number of exposed to non-exposed persons in the case group to the number of exposed to non-exposed persons in the control group; in cohort studies, OR is the ratio of the number of diseased to non-diseased persons in the exposed group to the number of diseased to non-diseased persons in the non-exposed group. The odds rate (OR) reflects differences in exposure factors between cases and controls, thus establishing an association between disease and exposure factors. The relative risk (RR) is usually the ratio of the exposed group’s incidence rate to the non-exposed group’s incidence rate (or a designated control group). During the meta-analysis, some scholars used the value of RR to analyze the relationship between exposure factors and disease, which had been indicated in the upper left corner with *. CI is the confidence interval, a statistical concept that a higher degree of confidence indicates that the results are more reliable. If OR or RR:

^a^ = 1, indicating no association between the exposure factor and the disease;

^b^ > 1, indicating that the exposure factor is a risk factor for the disease;

^c^ < 1, indicating that the exposure factor is a protective factor for the disease.

**Table 2:** Psychological disorders caused by prolonged or excessive interaction with cyberspace.

ICD-11	Cyber-Syndrome	MeSH	Indicator		Ref
			OR/R rate	95%CI	
Sleep-wake disorder	Sleep Initiation and Maintenance				
	Disorders (Insomnia)	D007319	1.18^*^	[1.01, 1.36]	^63^
	Narcolepsy	D009290	1.57^*^	[1.00, 2.46]	^64^
Mental, behavioural or neurodevelopmental disorders	Depression	D003863	1.60^*^	[1.45, 1.75]	^65^
	Anxiety Disorder	D001008	0.29	[0.23, 0.35]	^66^
	Obsessive-Compulsive Disorder	D009771	0.40	[0.22, 0.59]	^67^
	Somatoform Disorders	D013001	0.40	[0.20, 0.59]	
	Attention Deficit Disorder with Hyperactivity	D001289	3.76^*^	[2.75, 5.15]	^68^
	Anorexia Nervosa	D000856	2.03^*^	[1.58, 2.62]	^69^
	Bulimia Nervosa	D052018			
	Binge-Eating Disorder	D056912			

The relationship between exposure factors and disease was generally analyzed by R and OR during the meta-analysis, with OR already indicated by * in the upper left corner to make the distinction. The significance of OR is mentioned in the footnote of Table 1. The correlation coefficient R is often used to indicate whether there is a relationship between two events, which in this case represents the correlation between the disease and the causative agent. Assuming that two events are designated as X and Y, then if:

^a^R = 0, indicating that there is no correlation between X and Y;

^b^0 < R < 1, indicating a positive correlation between X and Y;

^c^-1 < R < 0, indicating a negative correlation between X and Y.

Apart from Table 1, which presents the set of physiological disorders obtained by meta-analysis, other pieces of evidence from the literature also provide the basis for physiological disorders due to excessive interaction with cyberspace. For example, the skin diseases it causes include erythema [D004890], acne rosacea [D012393], pruritus [D011537], seborrheic dermatitis [D012628], atopic dermatitis [D003876], acne vulgaris [D000152], etc.^5^ Mental, behavior, or neurodevelopmental disorders include mild cognitive impairment (MCI) [D060825], Alzheimer’s disease [D000544], amnesia [D000647], and so on^6^ Disorders of the visual system involve asthenopia [D001248], astigmatism [D001251]^7^ hyperopia [D006956]^8^ esotropia [D004948]^9^ etc. Disorders of the digestive system involve inflammatory bowel disease [D015212], constipation [D003248], irritable bowel syndrome [D043183], celiac disease [D002446] (https://www.bonum.lt/en/health/kaip-per-ilgas-sedejimas-veikia-jusu-zarnyna.html), dental caries [D003731], gingivitis [D005891]^10^ hemorrhoids [D006484]^11^ non-alcoholic fatty liver disease[D065626]^12^ dyspepsia [D004415]^13^ etc.. Disorders of the circulatory system include heart disease [D006331]^14^ of which heart failure [D006333]^14^ and coronary artery disease [D003324]^15^ are typical, and atherosclerosis [D050197]^16^ etc. Disorders of the nervous system involve tension-type headaches [D018781], migraine disorders [D008881]^17^ etc. There are also musculoskeletal disorders caused by prolonged and repetitive activities in cyberspace, such as back pain [D001416], neck pain [D019547]^18^ intervertebral disc displacement [D007405]^19^ kyphosis [D007738]^20^ sciatica [D012585]^21^ tennis elbow [D013716]^22^ cervical spondylosis [D055009]^23^ tenosynovitis [D013717]^24^ osteoarthritis [D010003]^25^ etc. Moreover, it can cause diseases of the ear or mastoid process, such as noise-induced hearing loss [D006317], etc.^26^ It can also cause endocrine, nutritional or metabolic diseases, of which obesity [D009765]^27^ hypercholesterolemia [D006937]^28^ and hyperuricemia [D033461]^29^ are typical. Furthermore, prolonged Internet access and accompanying behaviors such as sedentary and improper posture can increase the risk of neoplasms for users in cyberspace. The high-risk tumors include endometrial neoplasms [D016889]^30^, etc. Some cohort studies have revealed that those behaviors can also cause sleep-wake disorders, such as sleep apnoea [D012891], of which obstructive sleep apnoea [D020181]^31^ is a type, etc.

Table 2 outlines the psychological disorders derived from the meta-analysis, and evidence from other literature provides a rationale for psychological disorders arising from excessive interaction with cyberspace. For example, H. T. Vries *et al*.^32^ shows that pathological Internet use is associated with social anxiety disorder [D000072861] and autism spectrum disorder [D000067877]. M. Vismara *et al*.^33^ noted a significant correlation between cyberspace use and hypochondria [D006998].

So far, we have completed a synthesis of the various disorders encompassed by Cyber-Syndrome based on the results of the current literature. The Cyber-Syndrome corpus consists of texts from the Mayo Clinic (http://www.mayoclinic.org), one of the leading not-for-profit medical institutions in the United States and the world. For each Cyber-Syndrome, the Mayo Clinic website provides a text consisting of the following sections: overview, symptoms, causes, risk factors, complications, when to see a doctor, etc. We use the first three sections of each text. For the purpose of obtaining a text corpus for each Cyber-Syndrome, we exploited a web crawler, a program that automatically extracts data from websites. The text corpus for each Cyber-Syndrome was named with its unique MesH code. A total of 87 texts were gained, including 3,472 sentences and 42,964 words.

#### Acupoints

The concept of acupoints derived from *Huangdi’s Canon of Medicine (Huangdi Neijing)* and *The Great Compendium of Acupuncture and Moxibustion*. Acupoints are particular points on the body’s meridian lines where certain substances can be released or changes caused that can regulate the function of organs, maintain homeostasis or treat disease^34^. The benefits of the acupoints can be facilitated through mechanical stimulation of traditional needling, thermal stimulation of moxibustion, electrical stimulation of electro-acupuncture, and radiation stimulation of laser acupuncture. For example, results published in Nature by Ma Qiufu’s research team demonstrated that low-intensity needling stimulation of mouse hindlimb acupoints (e.g. Foot San Li ST36) can activate the vagal-adrenal anti-inflammatory pathway and perform effective anti-inflammatory functions, providing a modern neuroanatomical basis for the relative specificity of the acupoints^35^. Irritation of various acupoints activates the respective neural pathways, thus providing preliminary evidence for the existence of acupoints.

The corpus of acupoints in the CS-A corpus is derived from the TCM Wiki (https://tcmwiki.com/), hosted by Contabo GmbH. This website explains acupoints, herbs, and prescriptions in English as the primary language, covering a more comprehensive knowledge of TCM theory. For each acupoint, the TCM wiki provides sections on a general description, meaning, location, indication, acupuncture/moxibustion method, etc. We used an automated program to extract the corpus of the above sections from the website (i.e. a web crawler) and captured the text of 361 acupoints on the fourteen meridians (a general term for the twelve meridians along with conception vessel and governor vessel), individually named with the English names of the acupoints. The distribution of acupoints on each meridian is shown in Table 3. In total, the acupoint corpus includes 3,906 sentences and 33,063 words.

**Table 3:** Statistics on the distribution of acupoints.

Meridian	Abbreviations	Number of acupoints	Meridian	Abbreviations	Number of acupoints
Lung Meridian of Hand-Taiyin	LU	11	Large Intestine Meridian of Hand-Yangming	LI	20
Spleen Meridian of Foot-Taiyin	SP	21	Stomach Meridian of Foot-Yangming	ST	45
Heart Meridian of Hand-Shaoyin	HT	9	Small Intestine Meridian of Hand-Taiyang	SI	19
Kidney Meridian of Foot-Taiyin	KI	27	Bladder Meridian of Foot-Taiyang	BL	67
Pericardium Meridian of Hand-Jueyin	PC	9	Triple Energizer Meridian of Hand-Shaoyang	TE	23
Liver Meridian of Foot-Jueyin	LR	14	Gall Bladder Meridian of Foot-Shaoyang	GB	44
Conception Vessel	CV	24	Governor Vessel	GV	28

#### Diseases and symptoms lexicon

We used a dictionary-based approach to pre-annotate the disease, Cyber-Syndrome, acupoint, symptom, and sign mentioned in the CS-A corpus to improve annotation consistency and reduce the heavy workload of manual annotation. The Cyber-Syndrome dictionary and the acupoint dictionary have been given in the two sections above, respectively. This section presents the disease dictionary and the symptom/sign dictionary.

- Disease Dictionary: The Disease Ontology (DO)^36^, which contains 8075 diseases and semantically integrates medical vocabulary through extensive cross-mapping with MeSH, ICD, NCI’s thesaurus, SNOMED, and OMIM, has been developed as a standardized ontology for human disease and was created by the University of Maryland School of Medicine (Genome Sciences Institute). An automated procedure extracts the diseases contained in the DO to form a dictionary for pre-annotation work.
- Symptom/Sign Dictionary: The Symptom Ontology (SYMP), containing 893 symptoms or signs of disease, is a standardized symptom ontology of disease, developed by the University of Maryland (Genome Sciences Institute). The symptoms covered by the SYMP form a symptom dictionary which provides the foundation for pre-annotation tasks.

At present, the above-mentioned dictionaries (Cyber-Syndrome, acupoints, disease, and symptom) have been uploaded to the *figshare*^37^ database, and researchers in need can download them themselves. The automatic pre-annotation identified 1,456 diseases, 1,341 Cyber-Syndrome, 577 acupoints, and 2,446 symptoms/signs. Finally, the pre-annotated text was given to the human annotators as a starting point.

### Annotation Guidelines

The CS-A corpus is manually annotated using the Brat annotation tool. It is an intuitive web-based tool for text annotation running on Linux supported by NLP technology^38^. Brat is available under an open source license from: http://brat.nlplab.org. When using Brat for annotation, each sample file must have an ann file with it which is mainly used to store the annotation results generated automatically after annotation. Parsing of ann file can be used in named entity identification (NER) studies.

Multiple available corpora were referenced to design clear and accurate annotation guidelines. For example, the NCBI disease corpus^39^, a collection of 793 PubMed abstracts, was annotated primarily for disease mentions, with 2 annotators assigned to each document to ensure the accuracy of annotation. The NCBI disease corpus has eight annotation rules, and the annotation rules that are relevant to this paper are: ① Annotate disease mentions that match in the MEDIC or OMIM databases; ② If not available, annotate synonyms that match disease mentions; ③ Annotate the closest hyperbolic logical concept that correctly describes the disease mentions. ④ Annotate composite diseases with the “|” separator and multiple conceptual descriptions of the disease mention with the “ + ” operator; ⑤ Annotate disease mentions even if they are identical to the gene names. We also refer to the RareDis corpus^4^ which contains 1041 documents derived from the rare diseases database, created and maintained by the National Organisation for Rare Diseases (NORD). Four annotators have completed the annotation task. There are 11 annotation rules designed for the RareDis corpus, which inspired the annotation work in this paper. The DDI corpus^40^ corpus contains 1025 documents from the DrugBank database and Medline. This corpus focuses on the interaction between drugs, so when annotating, the mechanism, effect, advice, and int relationships between entities are collectively referred to as DDI relationships, simplifying the annotation task and providing new ideas for corpus annotation work.

Inspired by the corpus above, the annotator group defined the entity and relationship types to be annotated during the annotation specification phase. Meanwhile, the annotator group provided specific descriptions of the defined entities and relationship types and enumerated the relevant examples, which facilitated the annotator group to complete the annotation task accurately. The defined types are shown in Table 4, and the relationship types are shown in Table 5. Definitions of the sign, syndrome, and acupoint entity descriptions in Table 4 are from the National Cancer Institute (https://www.cancer.gov/). To annotate the CS-A golden corpus, annotator groups are encouraged to use their professional knowledge and public resource knowledge base. The following annotation specifications have been summarized.

**Table 4:** Description of the entities annotated in the CS-A corpus.

Entity Type	Description	Examples
Disease	The accumulation of a certain amount of pathogenic factors causes cell damage, and the damaged cells cause functional, metabolic, and morphological structural disorders, ultimately manifested as abnormalities in symptoms, signs, and behaviors.	*hirata disease, bruxism, lower lip cancer*
Cyber-Syndrome	A general term for various diseases that arise from users experiencing physical, psychological, and other discomfort due to prolonged interaction with cyberspace.	*myopia, autism spectrum disorder, diabetes mellitus*
Sign	Somethings found during a physical exam or from a laboratory test that shows that a person may have a condition or disease.	*inflammation, high heart rate, thrombocytopenia*
Syndrome	A physical or mental problem that a person experiences that may indicate a disease or condition.	*neck pain, numbness, blurred vision*
Acupoint	A specific spot on the body where an acupuncture needle may be inserted to control pain and other symptoms.	*Baihui, Chengfu, Jugu*
Anaphor	Generally represented by personal pronouns and demonstrative pounds, referring to antecedents.	*This disease, It*

**Table 5:** Description of the relationships annotated in the CS-A corpus.

Relationship	Description
Is_a	The relationship between a specific disease being one of the more general diseases
causes	The relationship between a sign or a symptom caused by a disease or a Cyber-Syndrome
Is_synon	The relationship between two different names designating the same disease^4^, Cyber-Syndrome, sign or Syndrome
Is_acron	The relationship between an acronym and full name of the disease or Cyber-Syndrome
Is_anaphora	The relationship between anaphor and its ancestors
treats	The relationship between the corresponding treatment symptom of the acupoint

#### Overlapped entity

NER is a very fundamental task in NLP, and the focus of NER research has gradually shifted from conventional flat-named entity recognition (Flat NER) to overlapped entity. Some mentions were annotated with two or more different entity types during the annotation process, which are called overlapping entities. It is quite common for overlapped entities to be found in BioNLP, so it is essential to annotate overlapped entities. Thus, the following rules are established for overlapped entities:

- If a mention can be annotated as both disease and Cyber-Syndrome, only the more specific entity is annotated, i.e. Cyber-Syndrome.
- If a mention can be annotated as either disease/Cyber-Syndrome or sign/syndrome, then the entity type is annotated according to the context of the sentence. If the context can not clearly express whether it is a syndrome/sign or disease/Cyber-Syndrome, the default is the disease/Cyber-Syndrome entity.

**Fig. 1:**
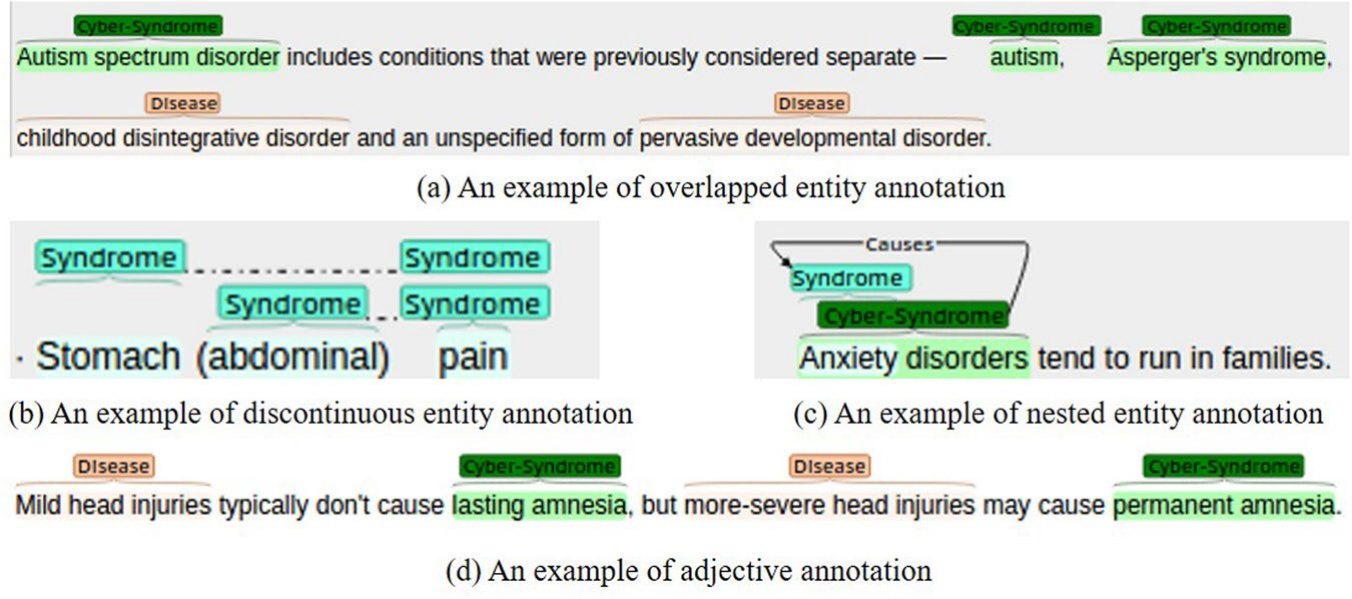
Examples of entity annotation: (**a**) overlapping entities; (**b**) discontinuous entities; (**c**) nested entities; (**d**) adjectives.

As shown in Fig. 1a, Autism is both a *Disease* entity, a *Cyber-Syndrome* entity, as well as a *syndrome* entity. According to the above annotation rules, Autism is annotated as a *Cyber-Syndrome* type.

#### Discontinuous entity

Discontinuous NER has always been a challenging task in NLP. Unlike conventional continuous named entities, the recognition of discontinuous NER faces two significant challenges. Firstly, there are intervals within discontinuous named entities. Secondly, discontinuous named entities are often accompanied by overlapping phenomena^41^. Therefore, the conventional named entity extraction methods are ineffective in extracting discontinuous entities and suffer from problems such as understanding ambiguity and low recognition success rate.

Overall, discontinuous NER datasets are scarce, with limited training data available compared to traditional NER tasks. The addition of discontinuous entity annotations to the CS-A corpus would increase the available data sources in this area. The mentions consisting of discontinuous spans are quite common in our corpus. Discontinuous entities also have plenty of critical information embedded in them. It is important to annotate discontinuous entities to improve the usefulness of the CS-A. Figure 1b shows how to annotate the discontinuous entity.

#### Nested entity

In the medical domain, nested entities are often occurring. Nested entities are rich in semantic relationships that are critical to the execution of downstream tasks. However, the structure of nested named entities is complex and variable, and the granularity of nesting and the number of nesting layers lack regularity. The key to the full-scale application of NLP is how to quickly and efficiently obtain accurate information about the structure of nested named entities from open domain texts to make semantic understanding more accurate.

Nested entities were annotated in the CS-A corpus to provide quality data for advancing nested entity naming recognition research. If the long entity is of a distinct type from the nested short entity, they are both to be annotated. For example, *social anxiety disorder*, which is a *Cyber-Syndrome*, contains *anxiety*, which is a syndrome. In this case, both entities were annotated. One particular case is when the syndrome/sign and the disease are of the same name, without nested annotation. Identify the type of *Syndrome/Sign* or *Disease* depending on the context. An example of the annotation is shown in Fig. 1c.

#### Adjective annotation

During the annotation process, it was found that adjectives preceded some mentions, and the adjectives should be annotated together with the entities. This is because these adjectives can indicate symptoms to varying degrees and the type of *disease/Cyber-Syndrome*. These adjectives include slight, mild, severe, significant, etc. The annotations are shown in Fig. 1d.

#### Relationship annotation

**Fig. 2:**
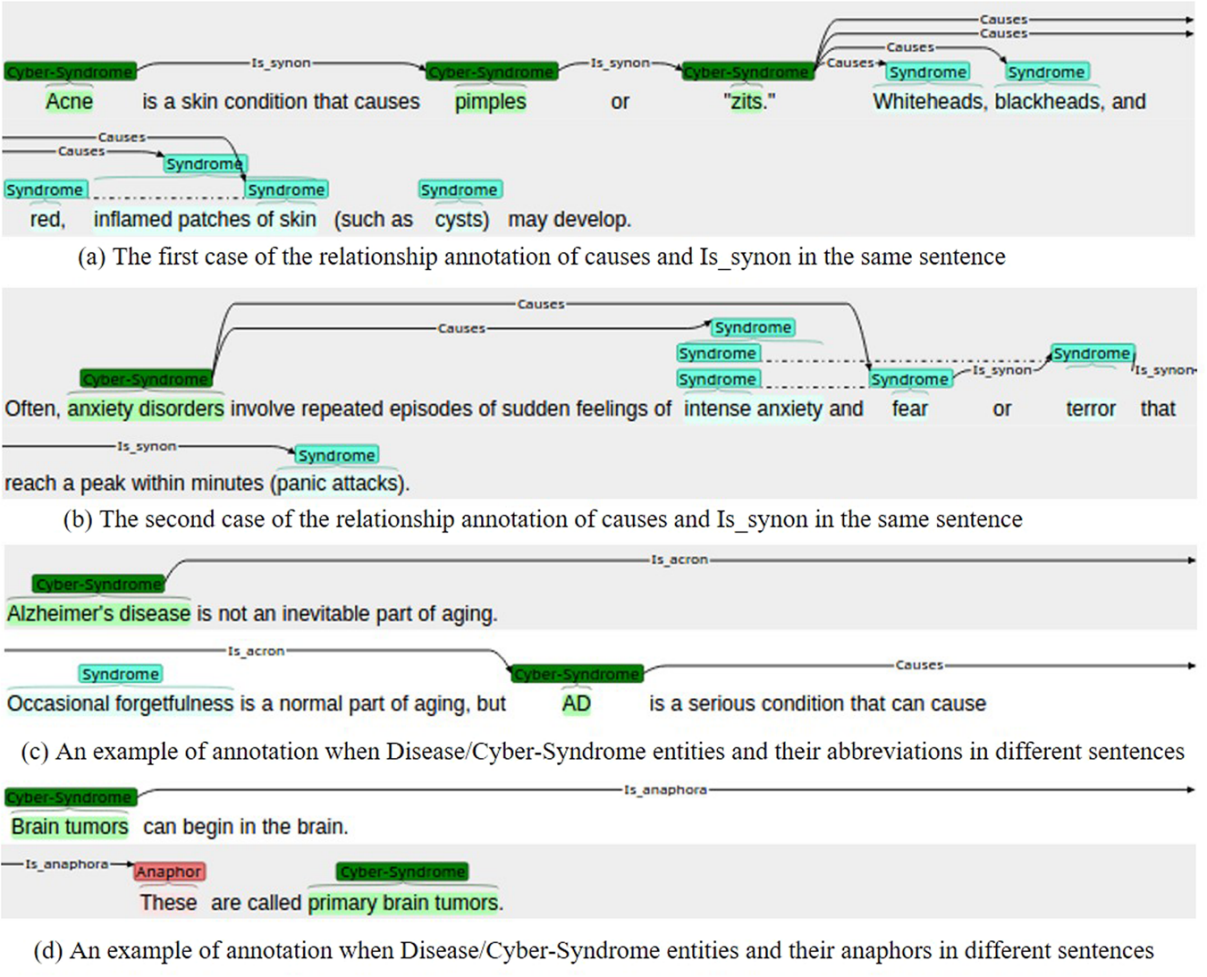
Examples of relationship annotation. (**a**) The first case of the relationship annotation of *causes* and *Is_synon* in the same sentence. (**b**) The second case of the relationship annotation of *causes* and *Is_synon* in the same sentence. (**c**) An example of annotation when *Disease/Cyber-Syndrome* entities and their abbreviations in different sentences. (**d**) An example of annotation when *Disease/Cyber-Syndrome* entities and their anaphors in different sentences.

The relationship should be annotated at the sentence level when multiple mentions appear in the same sentence. If several mentions of the same entity are mentioned in the same sentence, *causes* relation is annotated by the adjacency. There are two cases as follows. If there are no relationships between *syndrome/sign* in the same sentence, the *causes* relation is annotated to each *syndrome/sign* entity, as shown in Fig. 2a. If there are *Is_synon* relationships between the *syndrome/sign* in the same sentence, only the first *syndrome/sign* entity is annotated, as shown Fig. 2b.

The *Is_synon* and *Is_acron* relations are transitive. When there are at least three same mentions in a sentence, only the two neighboring identical entities will be annotated with the *Is_synon* and *Is_acron* relations, and no cross-entity annotation will be performed (Fig. 2c). Naturally, there are cases where entities are annotated with relations in different sentences. The details are as follows: (1) When the disease or Cyber-Syndrome entity and its abbreviation are in different sentences, the *Is_acron* relationship between them is annotated as shown in Fig. 2c. (2) When the disease or Cyber-Syndrome entity and its anaphor are in separate sentences, the labeling is shown in Fig. 2d.

## Data Records

The CS-A corpus primarily encompasses two file types, txt files that are not manually annotated, and ann files that are iteratively manually annotated (accomplished with the help of the brat annotation tool). Each txt file should correspond to an ann file, with 448 txt files and 448 ann files. Moreover, a tree structure was constructed for each Cyber-Syndrome and the acupoints that can alleviate its symptoms were stored in pdf files according to algorithms such as cosine similarity calculation and character matching. In total, 87 PDF files are available to show the tree structure of each Cyber-Syndrome. The CS-A tree corpus was also stored in a notepad storage format, making it easy for the researcher to choose which storage format to use according to their needs. The above collection of files have all been uploaded to *figshare*^37^.

## Technical Validation

### The CS-A corpus statistics

**Fig. 3:**
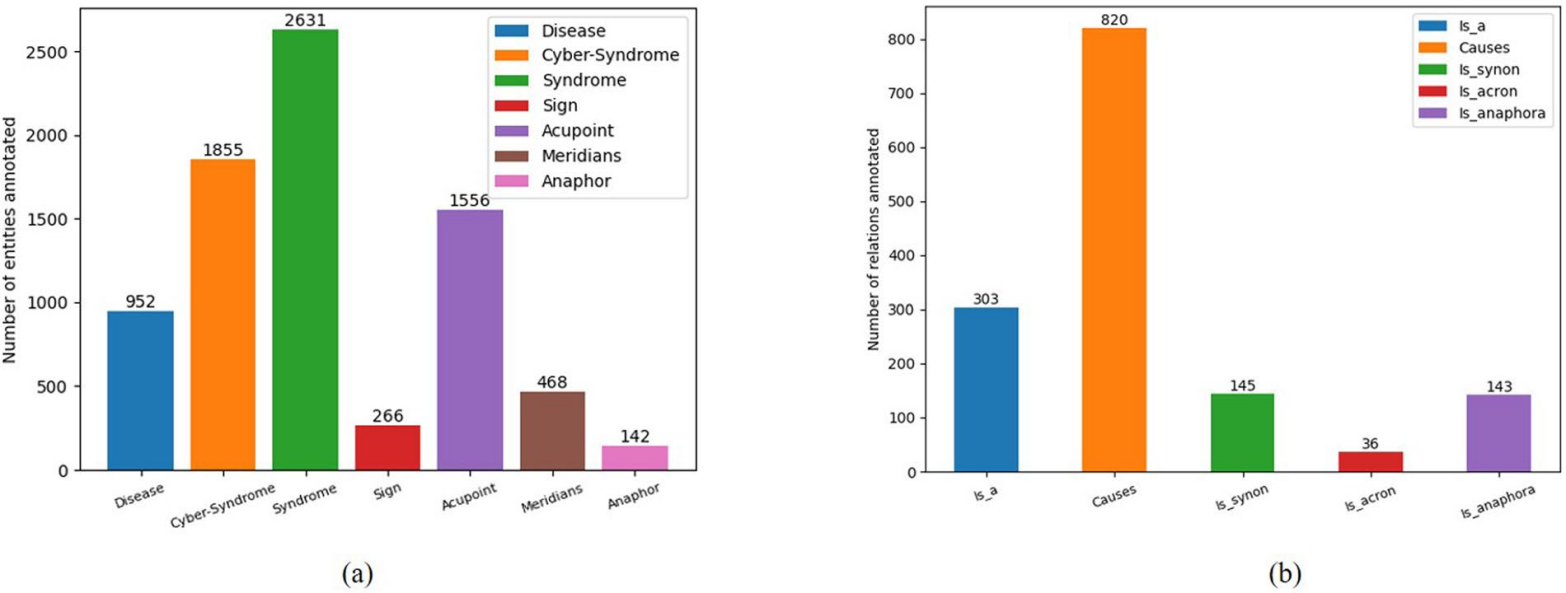
Statics on the ultimate number of entities and relationships annotated: (**a**) Number of entities (**b**) Number of relations.

There are two novel points in the CS-A corpus, one is the groundbreaking definition of Cyber-Syndrome and the types of diseases it encompasses, and the other is the provision of ideas for preventing and treating Cyber-Syndrome from the perspective of acupoints of TCM. This work could provide the BioNLP community with an annotated corpus for training and learning different machine-learning algorithms to extract valuable information from medical texts. We counted the number of texts, sentences, and words in the CS-A corpus with the help of nltk, a natural language processing toolkit. There are 448 texts, 7,378 sentences, and 76,027 words. The annotators manually annotated the pre-annotated text of the CS-A corpus, and the number of entities and relations annotated is shown in Fig. 3.

### Annotation consistency assessment

The performance of NLP algorithms is limited by the bias of different annotators’ data comprehension. Therefore, during the annotation process of building the corpus, Inter-annotation agreement (IAA) is used to evaluate the degree of agreement between independent annotators when annotating the same entity to ensure the quality of the corpus. The most straightforward and intuitive way to access consistency is to statistically count the percent of annotated samples that are consistent as a proportion of all annotated samples, and the F1-score measure does just that. Assuming that the results of the first annotator’s annotation are used as the standard, then we count the results of the second annotator and calculate the values of precision, recall, and F1-score with the formulas shown below:

*Let the set of samples annotated by annotator 1 as A, and the set of samples annotated by annotator 2 as B*. $A\cap B$ *yields the result of annotating the consistent items*.

1 $$Precision=\frac{count\left(A\cap B\right)}{count(B)}$$

2 $$Recall=\frac{count\left(A\cap B\right)}{count\left(A\right)}$$

3 $$F1-score=\frac{2\ast Precision\ast Recall}{(Presion+Recall)}$$

**Fig. 4:**
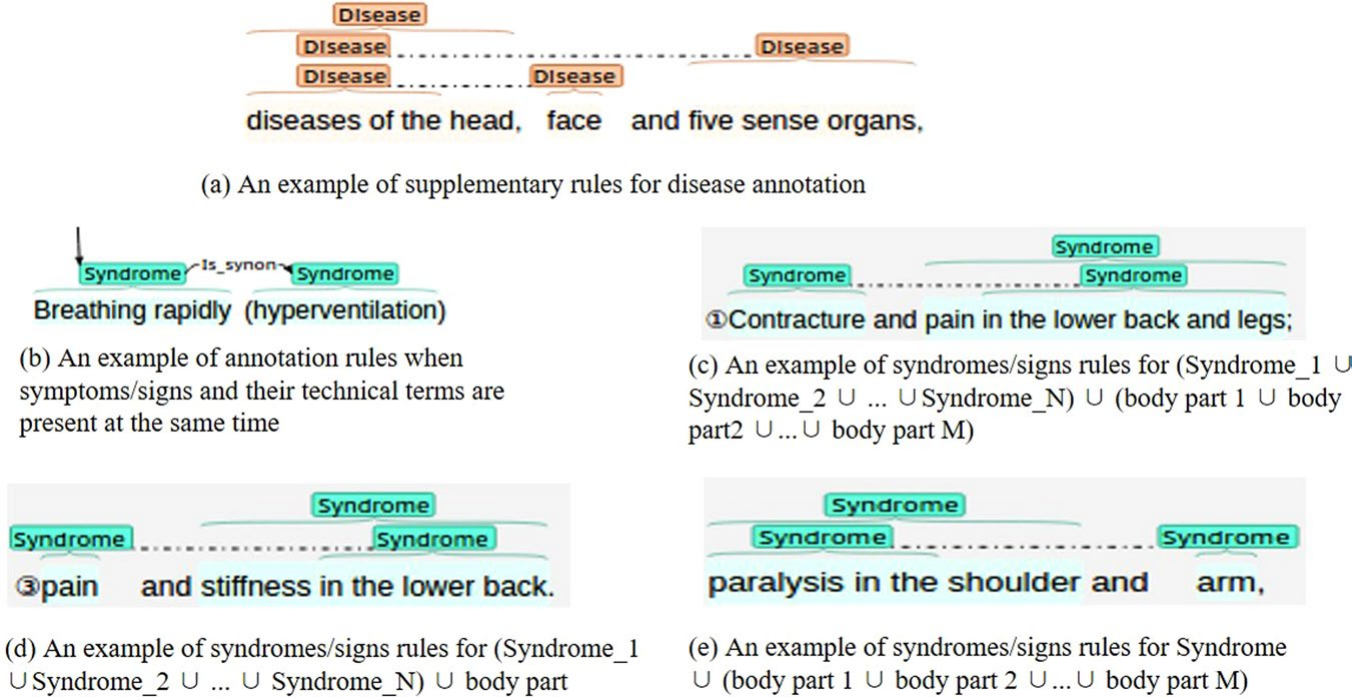
Examples of supplementary rules after inconsistency annotation analysis. (**a**) An example of supplementary rules for disease annotation. (**b**) An example of annotation rules when symptoms/signs and their technical terms are present at the same time. (**c**) An example of syndromes/signs rules for *(Syndrome_1* ∪ *Syndrome_2* ∪... ∪ *Syndrome_N)* ∪ *(body part 1* ∪ *body part 2* ∪...∪ *body part M)*. (**d**) An example of syndromes/signs rules for *(Syndrome_1* ∪ *Syndrome_2* ∪... ∪ *Syndrome_N)* ∪ *body part*. (**e**) An example of syndromes/signs rules for *Syndrome* ∪ *(body part 1* ∪ *body part 2* ∪...∪ *body part M*).
- Syndrome/Sign: Low syndrome/sign agreement was found during the inconsistency analysis, which further constrained the annotation guidelines. First, if both a succinct description of the syndrome/sign and its technical terminology appear in the corpus, then both are annotated. At the same time, there is an *Is_synon* relationship between the succinct description and its technical terminology. As the annotation example given in Fig. 4b. Next, When annotating syndrome/sign, if it is a grouping of *Syndrome_1* ∪ *Syndrome_2* ∪*…* ∪ *Syndrome_N* ∪ (body part 1 ∪ body part 2 ∪… ∪ body part M), then we consider multiple body parts as a whole, and then annotate each symptom and the whole separately, as shown in Fig. 4c. If it is a mix *(Syndrome_1* ∪ *Syndrome_2* ∪*…* ∪ *Syndrome_N* ∪ *body part)* combination, then we annotated each syndrome and body part separately, as shown in Fig. 4d. If it is a grouping of *Syndrome* ∪ *(body part 1* ∪ *body part 2* … *body part M)*, we annotate the syndrome and each body part separately, as shown in Fig. 4e.
- Relationships annotation: From the results of the first and second rounds of annotation in Fig. 5, the agreement of the annotation of the *Is_a* relation is relatively low. One reason is that there is a problem with limiting the relation annotation to a sentence-level understanding. What we need to make clear now is that the colon is followed by a complementary statement to what came before, then this is an entire sentence, and all the *Is_a* relations involved in it are to be annotated. In addition, it was found that the agreement of the *Is_anaphor* relation was not high. It was noticed that some annotators only focused on the existence of this relation between adjacent sentences, ignoring the fact that it also exists in the same sentences, so this ambiguity was corrected in the third round of annotation iterations. Finally, a higher agreement result was gained. The *causes* relationship, although higher than the other relationships, leaves much to be desired in terms of agreement. The *causes* relationship describes the causal relationship between the disease / Cyber-Syndrome and the syndrome/sign. Most of the disagreement is due to disagreement in the annotation of the syndrome/sign entities, i.e., improving the agreement in the annotation of the syndrome/sign entities would subsequently lead to an improvement in the agreement of the *causes* relationship. The same is true for the *Is_a* relationship, which will likewise be improved through the modification of the analysis as well as the improvement of the inconsistency guidelines for the annotation of the syndrome/sign entities.

Following the above formulas, we calculated each entity’s and relationship’s consistency. The first calculated IAA is 75.25%, indicating a low level of consistency in the annotated corpus. Since it is difficult to avoid human errors due to manual annotation, a second iteration of annotation was performed after cross-checking to improve the agreement to 82.17%. After that, the reasons for the non-consistency of the annotation results were generalized and analyzed. The details are as follows:

- General names of disease: General names of diseases are common in the CS-A corpus. However, the annotators handle it differently, which accounts for the inconsistency. Hence, we have clarified the rules for annotation of generic disease names, i.e. if an adjective appears (e.g., mental, gastrointestinal, etc.) before a generic term (e.g., disease, disorder, etc.), which gives valuable information about the disease, this disease mention will be annotated. Generic terms (e.g., disease, disorder, etc.) followed by a body part are also to be annotated. Not annotated if the disease or disorder occurs on its own. An example is presented in Fig. 4a.

**Fig. 5:**
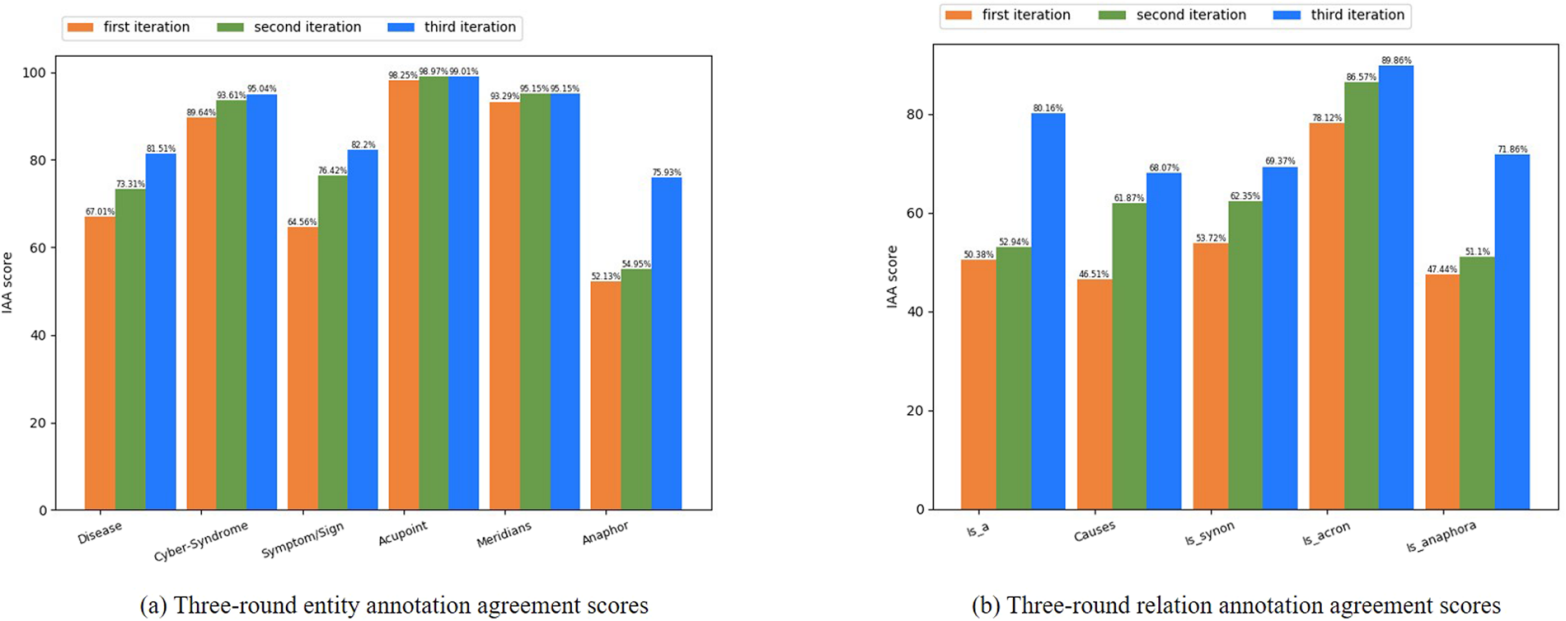
Comparison of annotation agreement scores for each entity and relation. (**a**) Three-round entity annotation agreement scores. (**b**) Three-round relation annotation agreement scores.

The final IAA value is 86.05%, which is a relatively high value for F1-score, representing the significance of the broad consistency of annotation among the annotators and the validity of the annotation guidelines mentioned in this paper, and can be used as a standard annotation guideline for the annotation of Cyber-Syndrome and the acupoints to prevent and control it. These high-quality annotation guidelines result in a higher quality of the CS-A corpus as well. Figure 5 compares the consistency of the three rounds of entities and relationships measured by IAA scores. The IAA scores show high agreement for Cyber-Syndrome (95.04%), acupoints (99.01%), meridians (95.15%), and moderate agreement for disease (81.51%), and symptoms/signs (82.06%). The reason for the relatively low agreement of diseases and symptoms/signs should be that there are inherent peculiarities of their specialties. Some disease mentions are medically ambiguous, with some categorized as clinical manifestations and others as disease types, which is a major cause of disagreement among disease entities. The disagreement that exists in the syndrome/sign is mainly because there are discontinuous entity annotations, and although the discontinuous entity annotations are regulated by guidelines, there are inevitably textual detail discrepancies. This also contributes to the low IAA score of 68.27% for *causes* relation. The lowest IAA score for anaphor (75.93%) is because anaphor generally relates to two types of entities, disease or Cyber-Syndrome, and while Cyber-Syndrome has a high degree of agreement, there is only 81.57% of agreement for disease, which can lead to lower scores for anaphor (typically less than the IAA scores for diseases). *Is_acron* (89.86%) has the highest IAA score for relations annotation because the relation between mentions and abbreviations is clearer. The remaining relations all received a significant increase in score after disagreement analysis.

### Tree corpus construction

**Fig. 6:**
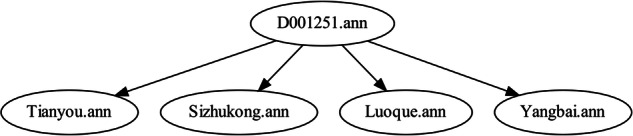
Example of CS-A corpus - Take the Cyber-Syndrome represented by D001251 as an example, the four acupoints of tianyou, sizhukong, luoque, and yangbai can relieve its symptoms.

Because long-term living in cyberspace puts people’s health at risk, the CS-A corpus was built to raise awareness of Cyber-Syndrome and to prevent and treat Cyber-Syndrome from the perspective of acupoints in TCM. Therefore, the CS-A corpus consists of the Cyber-Syndrome and acupoint corpus. To demonstrate which acupoints are better able to treat Cyber-Syndrome or alleviate the symptoms of Cyber-Syndrome, a hierarchical structure of trees was used to build the CS-A. The root node is the text corpus of Cyber-Syndrome and the leaf nodes are the text corpus of acupoints with which there is a therapeutic relationship. Perhaps there are no leaf nodes below the root node. In the CS-A corpus, 69.32% of the text corpus of Cyber-Syndrome has child nodes. Two methods were utilized to compute the child nodes, a string matching method for cases where the acupoints can directly treat the Cyber-Syndrome, and a cosine similarity to compute the phrase similarity for cases where the acupoints can alleviate the clinical symptoms presented by Cyber-Syndrome. After experimental validation, the second method has a similarity threshold of 0.8 to be recognized as a child node. There are a total of 1521 leaf nodes (i.e., *treats relations*). The resulting tree corpus hierarchy is shown in Fig. 6. This paper presents the structure of the CS-A in two forms, one in PDF form and the other in notepad form. That is, each Cyber-Syndrome and its acupoints that can treat or alleviate the symptoms are stored in a PDF or notepad file.

## Usage Notes

All data have been uploaded to *figshare*^37^. Both the original corpus and the manually annotated golden corpus were made available to the public. We hope that the corpus will draw the attention of researchers to the physical state of people when they are in cyberspace for long periods and that they will be able to explore different perspectives that can prevent Cyber-Syndrome from occurring. It also provides data to the BioNLP community to improve the efficiency of NLP techniques in processing information in fields such as medical information, and to solve the existing problem of difficulty in recognizing discontinuous entities, overlapping entities, nested entities, relationships, etc. Furthermore, the emergence of large language models (e.g., GPT3.5, GPT4, Llama, Qwen, Baichuan, etc.) now makes fine-tuning particularly important. In NER tasks, whether using LoRA, QLoRA, or instruction-based fine-tuning, it requires a manually annotated corpus. Moreover, when evaluating the model performance, a manually annotated corpus is also required to compute the evaluation metrics. In conclusion, the release of this corpus is of great significance and has a more considerable demand for application.

## Data Availability

Write data analysis code using Python and install packages such as Nltk, Numpy, and Pandas to assist. The code runs on the local computer. The data annotation in this paper uses the Brat tool (version: Brat-1.3p1), running on a Linux system. The code is mainly used for CS-A corpus generation and the quality analysis of the corpus. The code has been uploaded to the *GitHub* repository and is accessible using the following link: https://github.com/Xiduoduosci/CS_A_corpus.
